# Infrared Thermography Evaluation of Feet Temperature and Its Association with Claw Lengths and Anisodactylia in Purebred Sows of Three Greek Herds

**DOI:** 10.3390/vetsci8120309

**Published:** 2021-12-06

**Authors:** Fotios G. Kroustallas, Georgios A. Papadopoulos, Sofia Chalvatzi, Vasilis Skampardonis, Leonidas Leontides, Paschalis Fortomaris

**Affiliations:** 1 Laboratory of Animal Husbandry, Faculty of Veterinary Medicine, School of Health Sciences, Aristotle University of Thessaloniki, 54124 Thessaloniki, Greece; geopaps@vet.auth.gr (G.A.P.); schalvat@vet.auth.gr (S.C.); fortomap@vet.auth.gr (P.F.); 2 Department of Epidemiology, Biostatistics and Economics of Animal Production, School of Veterinary Medicine, University of Thessaly, 43132 Karditsa, Greece; bskamp@uth.gr (V.S.); leoleont@uth.gr (L.L.)

**Keywords:** infrared thermography, sow, claw length, anisodactylia

## Abstract

The aim of the study was to investigate the associations of lower feet temperature with claw lengths measurements in purebred sows. In total 22, 19 and 45 multiparous sows in three herds A, B and C of PIC, DANBRED and TOPIGS genetic lines respectively participated in the study. Mean parity was 2.5, 2.3 and 3.0 for sows from herds A, B and C respectively. Measurements were made during the periparturient period. Infrared temperature distribution was measured in carpus/tarsus, upper-lower metacarpi/metatarsi and phalanges (IRT1, IRT2, IRT3 and IRT4 respectively). In addition, dorsal, diagonal, heel–sole and dew claw lengths of medial and lateral claw were measured and the difference in dorsal claw length between medial and lateral claw (anisodactylia) was calculated in all four feet. Differences between herds regarding IRT and claw length measurements were analyzed with one-way ANOVA with herds as a fixed factor. Correlations between IRT and claw length measurements in each foot including data from all herds were evaluated using the Pearson’s correlation test. Maximum IRT1 to 4 in almost all rear feet, differed significantly between herds, being lower in sows of herd C than A and B (*p* < 0.05). Claw lengths of all feet were lower in herd C than those of A and B (*p* < 0.05). Anisodactylia, differed significantly only in rear feet between herds been higher in herd A than C and B (*p* < 0.05). In all sows, claw lengths and rear feet anisodactylia were positively correlated with maximum IRT1 to IRT4 (*p* < 0.05). According to the results, IRT temperature distribution of lower feet of purebred sows of different genetic lines were positive correlated with claw lengths measurements and anisodactylia. Collectively, measuring IRT temperature of lower feet of sows with mobile IRT device could be used as an additional tool towards monitoring feet and claw health.

## 1. Introduction

It is well established that the control of heat exchange between body surface and external environment plays a very important role in regulation of body temperature during different physiological phases and/or activities throughout life of homeotherms. Thermoregulatory adjustments can be induced not only by changes in environmental temperature but also by a variety of physiological situations including age, fasting and food intake, physiological stress circumstances [1]. This induces changes in internal temperature which are followed by changes in body surface temperature. The evaluation of surface temperature through infrared thermography represents a valuable tool to monitor the physiologic status, welfare and the stress responses of domestic animals [2]. The use of infrared thermography (IRT) is a non-invasive remote sensing method that can measure accurately surface temperature distribution variations [3]. In IRT, variations in temperature distribution are presented as shade gradients in images illustrating the emitted radiation from skin surface [4]. IRT has been broadly employed for diagnostic purposes in human and veterinary medicine [5,6,7]. With the use of mobile IRT cameras the changes in skin temperature distribution due to progress of inflammation in underlying tissues can be detected [6]. In most instances, heat increase is linked to inflammation, due to increased blood circulation and metabolism in tissues [8,9,10].

There is limited research evidence for the use of IRT in sows and its association with claw characteristics. It was reported that the IRT temperature distribution of tarsi, upper/lower metatarsi and phalanges of rear feet were increased in case of lameness in sows [11]. In the latter study, however, there was no investigation of the association between IRT of different anatomical sites of lower feet of sows with claw lengths and dorsal claw length difference between medial and lateral claw (anisodactylia). Lameness in sows has been associated with excessive claw growth [12,13]. Additionally, claw overgrowth was correlated with increased frequency of other claw lesions [14,15,16]. Anisodactylia was common in hyperprolific sows of modern genotypes [14]. According to Anil et al. [17] particular types of claw lesions were associated with a higher risk for lameness. It has been shown that in the case of overgrown claws, weight bearing distribution may shift towards the heel that may exacerbate claw lesion, claw horn integrity and claw health [18]. Recently, it was shown that an increase in heel–sole length decreased all mechanical indices of claws in sows [19]. Consequently, claw lesion, overgrowth and claw dissimilarity are the major reasons of underlying tissue damages. Therefore, it can be hypothesized that the use of mobile IRT cameras at herd level could detect any inflammation occurrence with temperature increment in sows. Indeed, in dairy cows IRT has been employed to detect hoof lesions and to an earlier diagnosis of laminitis [2,20,21]. More specifically, sole ulcers and hemorrhages were associated with a higher temperature at the area of coronary band [2,20,21].

Claw overgrowth may cause abnormal mechanical stresses in feet resulting in discomfort, tissue damage and localized increase in temperature suggesting inflammatory response. As claw overgrowth has been shown to differ between genetic lines, we hypothesized that similar differences may occur regarding their lower feet temperature. Hence, the objective of the present study was to measure temperature distribution in lower feet of front and rear feet in purebred sows by using IRT technologies and to investigate the possible associations between claw lengths and anisodactylia with IRT distribution in lower feet of purebred sows of different genetic lines.

## 2. Materials and Methods

### 2.1. Farms and Animals

The present study was conducted in three commercial indoor farrow-to-finish herds with an average crossbred sows inventory of 250 (Herd A), 350 (Herd B) and 370 sows (Herd C). The herds were situated in the Prefecture of Larisa (Farm A:39.490464, 22.351533 and Farm C:39.509933, 22.600897) and in the Prefecture of Kozani (Farm B: 40.206131, 21.974417). The purebred breeding sows belonged to PIC (Herd A), Danbred (Herd B) and Topigs (Herd C) genetic lines. Sows were reared throughout their productive life on comparable rations, housing and management practices in compliance with Council Directive 2001/88/EC. Before launching the study, the owners gave their oral consent for participation. No approval of the study protocol by an Animal Care Committee was required, because no action in the study was painful or invasive for the animals. The collaborating farms were participants together with the academic units in the “T1EDK -02073- FITSOW’ research project investigating the longevity and welfare of sows in commercial Greek farms (financed by the European Regional Development Fund of the European Union and by Greek national funds). The study lasted for 14 months, from March 2019 until May 2020. Neither the health status of the sows’ feet nor the reproductive performance was considered for the selection. We examined 22 (Herd A), 19 (Herd B) and 45 (Herd C) purebred, multiparous sows from the grant parent (GP) breeding stock population that were due to farrow during the aforementioned period. Mean parity was 2.5, 2.3 and 3.0 for sows from herds A, B and C respectively.

### 2.2. Housing and Management

All herds operated on weekly farrowing schedules. Two to three days’ post-weaning sows were moved to insemination barns where they were housed in individual stalls (60 cm width × 210 cm length × 110 cm height) on fully slatted concrete floors (Herds A and B), or solid concrete in the front and slatted concrete in the rear half floor (Herd C), with slats width 8 cm and slots opening 2 cm. Feed was offered twice daily (at 07:00 and 20:00 h), with an automatic delivery system equipped with individual drop feed boxes connected to feed line that automatically placed the feed in the sows’ metal feed trough (15 cm width × 35 cm length) and subsequently refilled the containers every 12 h. Sows had free access to drinking water through a nipple water drinker in the feeders.

Four weeks after insemination all pregnant sows were moved to gestation facilities according to the herd management operating program. In herds B and C sows were loose housed in gestation pens (400 cm × 400 cm), in static groups of five animals on partially (50–50) slatted/solid concrete floors without beddings, had free-access to individual feed troughs (15 cm width × 35 cm length) in non-locking stalls and ad libitum drinking water through nipple water drinkers. In herd A sows were loose housed in dynamic groups of 40–45 animals, in pens (1000 cm × 900 cm) on partially (50–50) slatted/solid concrete floors with no beddings, had free-access to nipple drinkers and to two automatic electronic sow feeders (ESF), located in the center of each pen. Feed was provided twice daily (at 07:00 and 20:00 h) in herds B and C with feed drop boxes delivery system. In herd A sows had free 24-h access to automatic feeders and feed delivery was controlled individually by a fully computerized feeding management software program. Forced ventilation system with side wall cooling/heating panels were used in insemination and gestation houses of all three herds to control ambient temperature in intended level (18 °C to 24 °C) with temperature sensors located in the middle of the houses 160 cm above the floor.

One week before the expected farrowing day (at 107th day-in-pig), pregnant sows were moved into farrowing barns where they remained until their weaning (26, 27 or 28 days’ post-partum for herds A, B and C, respectively). Farrowing facilities consisted of mechanically ventilated, thermostatically controlled farrowing rooms. Low and high ambient temperatures (20.0–25.0 °C) were monitored and controlled for each farrowing room during lactation using a ventilation control system located outside of the farrowing room and temperature sensor located in the center of each farrowing room, approximately 150 cm above the floor. Each farrowing room contained 8–10 farrowing units, consisting of farrowing pens with crate (75 cm width × 240 cm length × 105 cm height) and finger bars extending downwards on each side, located on a totally perforated plastic floor, equipped with solid rubber pads (75 cm × 40 cm) and plastic feeder for piglets under a heating lamp in the front site of the pen. Room temperature was maintained at 20 °C before farrowing, increased to 26 °C for the first 2 weeks of lactation and subsequently lowered to 22 °C. Feed in herds A and C were provided 2 times per day, manually (at 09:00 and 20:00 h) and sows had free access to drinking water through a nipple drinker in feeder. In herd B the farrowing stalls were equipped with electronic sow feeder and sows had 24-h access controlled from a computerized management feeding program according to a pre-determined lactation feeding curve and free access to a nipple water drinker in the feed trough.

### 2.3. IRT Measurements

**Figure 1:**
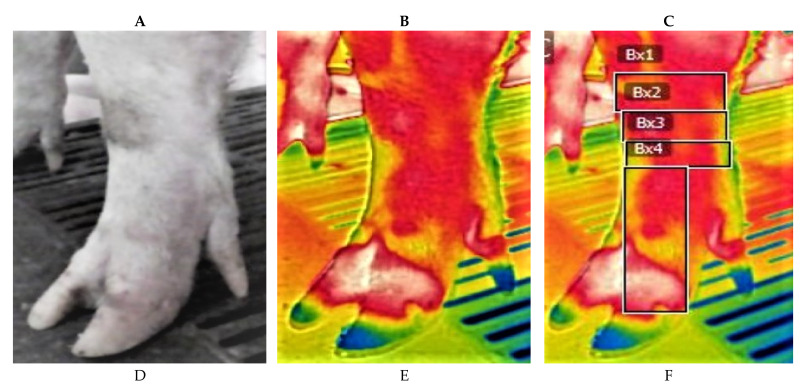

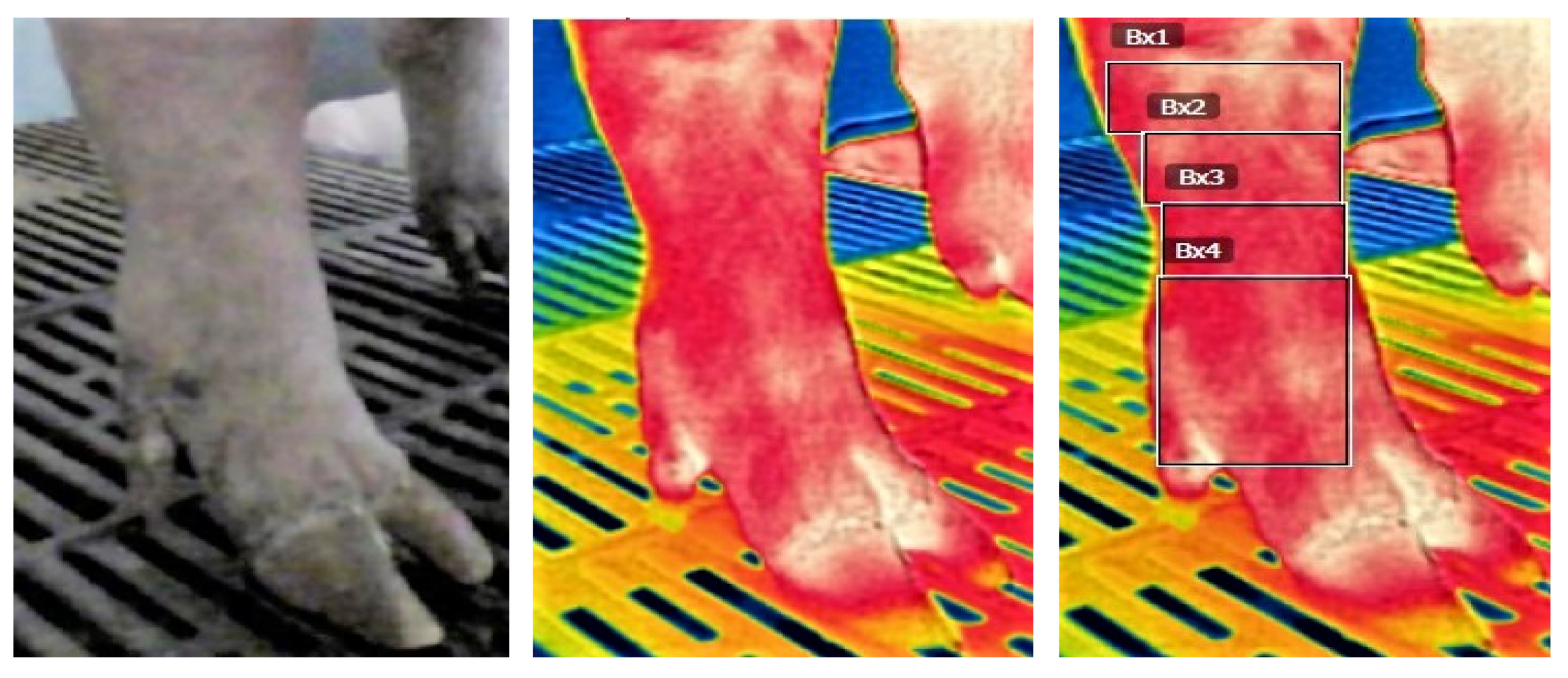
Analog and thermograme view of the regions of interest of one front and the opposite rear foot of two sows. (**A**): Analog view of front left foot; (**B**): Thermograme of front left foot; (**C**): Regions of interest, Bx1 Carpus area, Bx2 Upper Metacarpus, Bx3 Lower Metacarpi, Bx4 Phalanges. (**D**): Analog view of rear right foot; (**E**): Thermograme of rear right foot; (**F**): Region of interest, Bx1 Tarsus area, Bx2 Upper Metatarsi, Bx3 Lower Metatarsi, Bx4 Phalanges.

The last week of lactation, thermal images of sows’ lower feet were obtained by the principal investigator by using a high-resolution, long-wave thermal camera (FLIR E x8 Wi-Fi camera, Serial No 639040830, FLIR Systems Estonia, 7.5–14.5 μm, thermal sensitivity <0.06 °C, IR resolution 320 × 240, Accuracy ±2%, Operating T° −20–250 °C), 1–2 h post morning feeding of each farm. Thermograph resolution was calibrated to ambient temperature and humidity before each collection session. The emissivity value (e) which refers to the object’s ability to absorb and emit infrared radiation was set to 0.98 which is human skin emissivity as it refers to other similar studies [22]. In practice, emissivity is usually assumed to be in the range 0.98–1.0, which will have a minimal impact on temperature accuracy (<0.5 °C) [7]. Before measurement, each foot was cleaned out from fecal and dirt, using a dry cloth to minimize interference in IRT temperature. Prior to IRT scan, an acclimatization period of 4–5 min in standing position of sows were conducted. IRT images were captured at a focal distance of 70 cm–90 cm, while the sow was in standing position in farrowing stall avoiding direct sunlight and airflow. In addition, the span of the thermal camera and temperature range of color thermal scale was adjusted so the warmest region of the image appeared white or red and the coolest blue or black, thus maximizing the visual comparison of the thermal image. The acquired IRT thermal images of distal foot, enabled the measurement of skin surface temperature distribution for detecting localized abnormalities. The images were recorded as infrared image files, stored in camera’s memory and were transferred from the camera to a computer using the FLIR Quick Report 1.2 SP software program for image analysis. Quick Report 1.2 SP permitted to select regions of interest (ROIs), using a geometric figure drawn on the desired area. The software program allowed the user to display the temperature at any given ROI and calculated the maximum temperature (Figure 1).

### 2.4. Claw Length Measurements

**Figure 2:**
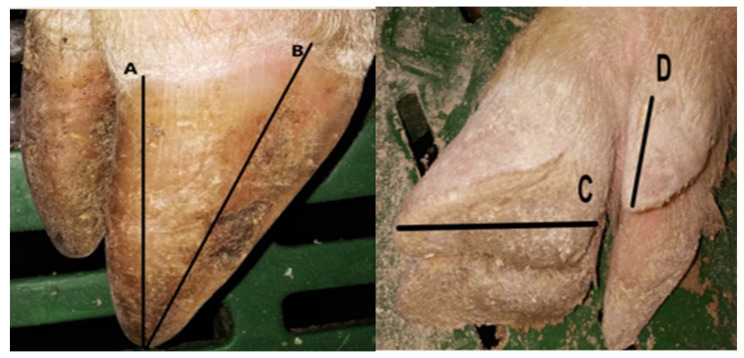
Claw length measurements: Dorsal length (**A**); Diagonal length (**B**); Heel–sole length (**C**); Dew claw length (**D**).

On 4th–5th day post-farrowing claw lengths of sows were measured with absolutely compliance to welfare protocol issues. All data were recorded on data forms along with ID number of sow, parity, date of farrowing, number of live born and dead piglets. Specifically, the length of the lateral and the medial claws and dew claws of each foot were measured, using electronic calipers (Facom 150 mm Digital Caliper 0.01 mm) at the following anatomical sites (Figure 2):

Dorsal claw length, along dorsal wall from just below the coronary band to the end of the wall (A). 

Diagonal claw length, along abaxial wall from the bottom of the wall at the toe to the top of abaxial wall–heel junction (B).

Heel–sole length, along palmar or plantar surface from the top of the toe to the caudal end of the heel (C). 

Dew claw length along dorsal wall from just below the coronary band to the end of the wall (D).

### 2.5. Statistical Analysis

Data was analyzed using the Statistical Package for the Social Sciences (SPSS) software (SPSS 25.0 Version, Chicago, IL, USA). Statistical significance was considered at *p* < 0.05. Differences between herds regarding IRT and claw length measurements were analyzed with one-way ANOVA of GLM procedure of SPSS with herds as a fixed factor and post-hoc comparisons between herds were made by Tukey’s test. The Kolmogorov–Smirnov test was used to investigate normality and Levene’s test to determine equality of variance. Results are presented as mean ± standard deviation (SD). Correlations between IRT and claw length measurements in each foot including data from all herds were evaluated using the Pearson’s correlation test.

## 3. Results

### 3.1. Maximum IRT Measurements

The maximum temperatures as depicted in IRT captures (mean ± SD) in the four anatomical regions of each lower feet are shown in Table 1. Maximum IRT1 and IRT2 of front and rear right foot, significantly differed between herds being lower in herd C compared to herd A and B (*p* < 0.05). Maximum IRT3 and IRT4 for both left and right rear feet differed between herds being lower in herd C, while maximum IRT4 in front left foot differed between herds being lower in herd C compared to A and B (*p* < 0.05).

### 3.2. Claw Length Measurements and Anisodactylia

The mean values (±SD) of claw lengths and dorsal length difference between medial and lateral claws (anisodactylia) in front and rear feet are shown in Table 2 and Table 3 respectively. Lateral and medial claw and dew claw lengths of almost all four feet, with the exception of front right medial dew claw length, significantly differed among herds being lower in herd C compared to those of A and B (*p* < 0.05). The dorsal length difference between lateral and medial claw (anisodactylia), only in both left and right rear feet differed among herds, being higher in herd A compared to herds B and C (*p* < 0.05).

### 3.3. Correlations between Maximum IRT Temperatures and Claw Lengths and Anisodactylia

**Figure 3:**
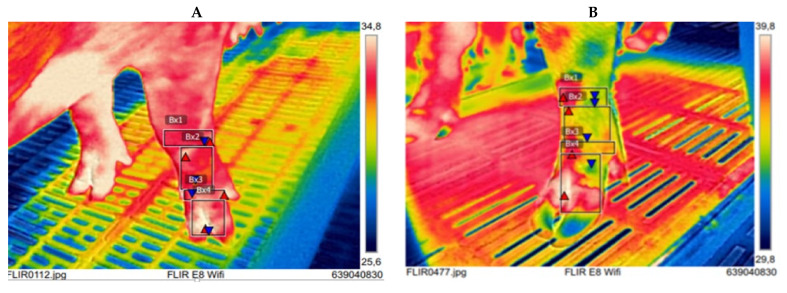
Infrared thermograms of the front left foot of two sows that differ in dorsal claw lengths with mild anisodactylia. IRT1 (Bx1); IRT2 (Bx2); IRT3 (Bx3); IRT4 (Bx4). (**A**) medial dorsal length 41.03 mm; lateral dorsal length 43.32 mm; Anisodactylia 2.29 mm; (**B**) medial dorsal length 59.66 mm; lateral dorsal length 61.64 mm; Anisodactylia 1.98 mm.

**Figure 4:**
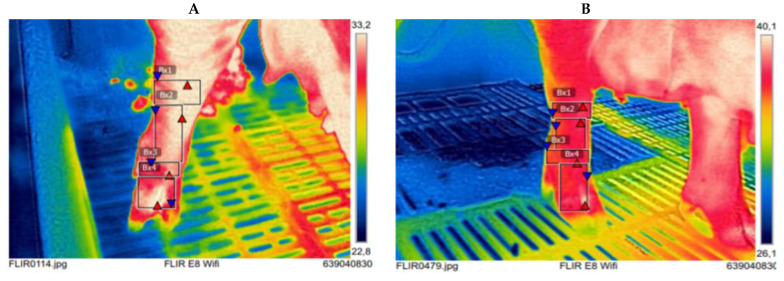
Infrared thermograms of the rear right feet of two sows that differ in dorsal claw lengths with severe anisodactylia. IRT1 (Bx1); IRT2 (Bx2); IRT3 (Bx3); IRT4 (Bx4). (**A**) medial dorsal length 46.84 mm; lateral dorsal length 55.45 mm; Anisodactylia 8.61 mm; (**B**) medial dorsal length 63.87 mm; lateral dorsal length 71.14 mm; Anisodactylia 7.27 mm.

Maximum IRT1, IRT2, IRT3 and IRT4 were positive correlated with claw lengths in almost all four feet in both medial and lateral claw, except IRT1 and IRT3 with heel—sole length of rear right and left medial claw respectively. In addition, positive correlations were observed between IRT1, IRT2, IRT3, IRT4 and anisodactylia only in almost all rear feet, with the exception of IRT3 and rear left foot anisodactylia (Table 4). In Figure 3 the differences in view of thermograms between two front left feet of two sows differed in claw length and anisodactylia are shown and in Figure 4 similar differences between two rear right feet are shown.

## 4. Discussion

Lameness in sows is a painful condition and welfare concerns are raised in the case of lame sows [23]. Additionally, hoof structure abnormalities such as overgrowth (dew)-claws and differences in length between medial and lateral claws have been associated with claw lesions and lameness [12,24]. It has been shown that the dissimilarity and length of claws become larger the frequency of claw lesions increases [16]. Therefore, it is necessary under field conditions to detect sows with compromised claw health and prevent lameness. For this reason, we have used mobile infrared cameras to assess temperature distribution in sows’ lower feet and to investigate the relationship between temperature and claw length measurements. Results of the study reveal that temperature distribution in lower feet of front and rear feet in purebred sows and claw lengths differ between herds and that longer claws are associated with increased IRT measurements.

Earlier reports suggested that purebred sows had reduced lifespan within the herds compared to crossbred sows, as they completed on average 4.4 litters than 5.3 litters till culling respectively [25,26]. In accordance, Jorgensen [27] showed that mean age and number of litters at removal were lower in purebred than crossbred sows. Interestingly, in the latter study the purebred sows had higher culling frequency for locomotion issues and reproductive failure [27]. For this reason, we focused firstly our investigation on purebred sows of three different genetic lines commonly used in commercial farms in Greece and elsewhere. Based on the findings of the study, it would worth to extend further the study on crossbreed gilts and sows. Usually claw lesions and sow lameness identified by visual observation in case of obvious abnormalities. Meanwhile, claw lesions and claw lengths are not frequently estimated, because it is a time consuming and labor-intensive procedure. Thus, it is possible that only sows with severe claw lesions and clinical signs of lameness can be usually detected, while those with compromised claw condition and subclinical lameness may not be spotted. For this reason, an early detection of inflammatory process before the onset of clinical claw lesion and lameness occurrence would be of practical importance for the pig industry [11]. In our study, the measured IRT temperature distribution in sows of three different herds, revealed a significant differentiation between the investigated genetic lines. It should also be noted that maximum IRT temperatures in the four regions of interest (IRT1, IRT2, IRT3, IRT4) were higher in the rear than front feet in sows. To our knowledge, only the study of Amezcua et al. [11] has demonstrated an alteration in IRT temperature differences between healthy and affected rear feet, implying that IRT thermal variation could be correlated with lameness in sows. In the latter study, IRT temperature in the areas of tarsus, upper metatarsi, lower metatarsi and phalanges of rear feet increased with lameness severity. Nevertheless, IRT was only measured in the rear feet of sows and not in all four feet. Driven by these research findings, we have extended the IRT investigation in all four feet. The region of interest was the area of phalanges, upper and lower metatarsi/metacarpi and tarsus/carpus. These anatomical areas are more vascularized than phalanges and hence changes in blood flow and local metabolism rate may be more evident via IRT [13,21]. Interestingly, the IRT values of the rear feet between our findings and that study displayed a difference of approximately 2.5–3 °C, being higher in our study than Amezcua et al. [11]. These findings are reasonable because we measured maximum temperatures and not the average in the region of interest. It is likely that there may be individual animal variations that can change at different times of the day affecting the skin surface temperature distribution [21]. Moreover, in our study IRT images were taken without prior foot washing because it was not able to wash in farrowing stalls. The higher IRT temperatures detected in the rear feet of sows compared to front feet could be attributed to weight bearing distribution. The weight distribution of sows is an important factor for the development of claw lesions in different feet [17,28]. It was suggested that lesions may not develop equally on all claws [17]. The latter authors proposed that the apparently healthy rear feet may have been affected by a subclinical disease process [17]. Thus, it can be hypothesized that sows may exhibit higher IRT temperatures in rear feet even without clinical claw lesion occurrence. The skin surface temperature demonstrated an individually contralateral symmetry between feet depended on underlying blood flow, tissue metabolism rate and may influenced by environmental conditions. IRT is a reliable and sensitive method facilitated the finding of regions with different skin surface temperature distribution. IRT may not always provide specific pathology details; however, it may assist definition and localization of inflammation areas and can be a useful diagnostic tool for early detection of asymmetry in skin surface temperature distribution between healthy and affected foot. In horses for several years IRT was used to identified lameness associated with inflammatory conditions [9,29]. In addition, in dairy cattle, IRT has been used to detect infectious diseases, laminitis and mastitis [2,8]. In horses, thermography can be used to detect signs of inflammation in the distal parts of the limbs before lameness is clinically evident [3]. In dairy cows, the healthy rear feet had higher IRT temperatures than healthy front feet [21].

Our study showed that the rear feet were the major location of overgrown claws with concurrent anisodactylia. This is in accordance with findings in other studies that reported higher claw lengths in rear feet than in front ones and in lateral claws than in medial ones [14]. Newman et al. [30], reported for the rear feet claw length values ranging from 51 mm to 79 mm (median 59.5 mm) and 38 mm to 50 mm (median 49.5 mm) for overgrowth and normal claws respectively, which are close to our findings. Sasaki at al. [16] reported toe length values from 46.20 mm to 46.60 mm and from 45.0 mm to 45.1 mm for lateral and medial claw respectively in a study concerning only the rear feet of gestating sows. In the study by Fitzgerald et al. [31] sows classified in the overgrowth group, were those with toe length greater than 63 mm. According to our findings, sows from herd A could be characterized as having overgrown claws especially compared to those from herd C. We have measured dorsal, diagonal and heel–sole length in all eight claws and dew claw length. The rear feet were the major location of overgrown claw and anisodactylia. This is in accordance with findings in other studies that reported claw lengths higher in rear feet than front and in lateral than medial claw. Fick [32] reported lower than our dorsal and heel–sole claw lengths values in sows both in lateral and medial claw. In addition, rear feet claw lengths were higher than front feet and lateral than medial claw with anisodactylia more pronounced on rear than front feet, in accordance with our findings. Mean growth rate of claw of 6.3 mm/30 days to 11 mm/28 days reported for gilts and sows respectively in recent studies [33,34] and while wear rate minimized to 5 m/30 days, unavoidable claw length continuously increased and diminishes with age [34]. Growth and wear rate are higher for the claws in rear feet and in lateral claws there are difference growth rate by breed and age. Yorkshire sows’ lateral toes growing more slowly when compared to Duroc and Crossbred (Duroc × Yorkshire) sows and parity two sows’ lateral toes growing the fastest, followed by parity one and three [34]. Newman et al. [30] evaluated histopathologic lesions among pig with overgrowth claws, used multiparous crossbreeding F1 sows (multiparous ones), reported for the rear feet claw length values of 51 to 79 mm (median 59.5 mm) and 38 to 50 mm (median 49.5 mm) for overgrowth and normal respectively claw (Overgrown defined claw length >50 mm), proximate to our findings. Interestingly, high toe lengths values were reported in a study of Fitzgerald et al. [31], in which 20 of 22 sows classified in overgrowth group (toe length >63 mm) having toe length between 64 mm and 102 mm and 76 of 84 sows classified in claw differences group (difference between medial and lateral claw >13 mm) having differences between 13 mm and 19 mm. These values concerning claw lengths are comparable and closer to our findings, but more different than previous mentioned studies. Causes of claw lesions and abnormalities are multiple and include toxic, parturition associated, hereditary and nutritional factors and may be affected by management, housing, body weight, weight distribution on individual claw, trauma, infection or a combination of these. The discrepancy in the size of the claws on the rear feet as well as the distribution of claw horn lesions indicate that the outer claw of the rear leg carries more weight relative to the inner claw [33] predisposing to inflammation process with increased skin surface temperature distribution due to mechanical insult.

In the current study, a positive correlation existed between maximum IRT and almost all measured claw lengths, both in medial and lateral claw, of all four feet of sows. In addition, anisodactylia in rear feet was positively correlated with maximum temperature of feet. The nature of these relationships can be explained by our recent findings that showed a positive correlation between overgrown claws and claw lesion frequency in sows [14]. It was also reported that claw mechanical efficiency deteriorated in the case of overgrown claws and higher claw lesion scores [19]. Although we have not assessed claw lesion severity in sows of the current study, it can be hypothesized that in longer claws the IRT would also be increased, probably because of ongoing inflammation processes caused by claw lesions. Varagka et al. [35] observed histopathological changes in sow’s feet, similar to those described in cases of equine and bovine laminitis. In the latter study other histopathological changes such as hyperemia, hemorrhage and edema were identified, which were associated with claw lesions of investigated feet. These findings are in accordance with the increased IRT measurements described in our study. Most likely, the initial histopathological changes due to acute phase of inflammation may have been responsible for increased temperature detected in IRT measurements. The occurrence of a subclinical laminitis in sows prior to a symptomatic phase and lameness occurrence could have also co-existed in those sows with overgrown claws and higher IRT recordings. This field warrants further investigation.

## 5. Conclusions

According to our results, IRT temperature distribution of lower feet of purebred sows of different genetic lines were positive correlated with claw lengths measurements and anisodactylia. Collectively, measuring IRT temperature of lower feet of sows with mobile IRT device could be used as an additional tool towards monitoring feet and claw health. Further research is needed towards validating the current findings, with particular focus on crossbreed gilts and sows which constitute most of the female reproductive population in pig herds and further investigation of association of IRT measurements with lameness occurrence.

## Figures and Tables

**Table 1:** Maximum temperature (mean ± SD) of each foot at four anatomical regions in purebred sows of all three herds.

	Herds			
	A	B	C	*p*-Value
	*n* = 22	*n* = 19	*n* = 45	
Max IRT1				
Front right foot	35.91 ± 1.59 ^a^	36.25 ± 0.88 ^a^	35.01 ± 1.80 ^b^	0.009
Front left foot	35.66 ± 1.57	35.37 ± 1.56	34.98 ± 1.80	0.293
Rear right foot	37.06 ± 1.24 ^a^	36.87 ± 0.82 ^a^	35.96 ± 1.44 ^b^	0.002
Rear left foot	36.78 ± 0.96	36.59 ± 1.11	36.06 ± 1.56	0.087
Max IRT2				
Front right foot	35.69 ± 1.70 ^a^	35.67 ± 0.90 ^a^	34.52 ± 2.15 ^b^	0.017
Front left foot	35.37 ± 1.82	35.02 ± 1.62	34.64 ± 2.01	0.326
Rear right foot	36.76 ± 1.27 ^a^	36.69 ± 0.79 ^a^	35.81 ± 1.44 ^b^	0.005
Rear left foot	36.58 ± 0.99	36.50 ± 1.09	35.84 ± 1.74	0.085
Max IRT3				
Front right foot	35.37 ± 2.03	34.74 ± 1.63	34.33 ± 2.37	0.183
Front left foot	35.34 ± 1.87	34.44 ± 1.77	34.32 ± 2.36	0.174
Rear right foot	36.55 ± 1.18 ^a^	36.34 ± 0.72 ^a^	35.26 ± 1.78 ^b^	0.001
Rear left foot	36.20 ± 1.00 ^a^	36.25 ± 1.04 ^a^	35.37 ± 1.79 ^b^	0.033
Max IRT4				
Front right foot	36.58 ± 1.55	36.17 ± 1.53	35.46 ± 2.84	0.162
Front left foot	36.96 ± 1.43 ^a^	35.7 ± 1.74 ^b^	35.43 ± 2.85 ^b^	0.045
Rear right foot	37.72 ± 1.39 ^a^	37.32 ± 0.86 ^a^	36.16 ± 1.73 ^b^	<0.001
Rear left foot	37.72 ± 0.84 ^a^	37.23 ± 1.06 ^a,b^	36.26 ± 1.76 ^b^	<0.001

Max IRT1: Maximum temperature at the joint of carpus in front legs or tarsus in rear legs; Max IRT2: Maximum temperature at the upper metacarpus or metatarsus area; Max IRT3: Maximum temperature at the lower metacarpus or metatarsus area; Max IRT4: Maximum temperature at the phalanges of each foot. ^a,b^ Mean values within the same row with different superscript letter are significantly different (*p* < 0.05).

**Table 2:** Claw lengths (mean ± SD) of medial and lateral claw of each front foot in purebred sows of all three herds.

	Herd			
	A	B	C	*p*-Value
	*n* = 22	*n* = 19	*n* = 45	
Front right foot				
Medial claw lengths (mm)				
Dorsal	51.92 ± 12.84 ^a^	53.34 ± 8.20 ^a^	43.94 ± 4.09 ^a^	<0.001
Diagonal	65.45 ± 14.73 ^a^	66.15 ± 8.84 ^a^	55.73 ± 4.58 ^b^	<0.001
Heel–sole	66.61 ± 14.97 ^a,b^	70.59 ± 8.13 ^a^	65.94 ± 6.06 ^b^	0.050
Dew claw	37.29 ± 11.32	38.30 ± 6.93	34.93 ± 5.16	0.327
Lateral claw lengths (mm)				
Dorsal	53.29 ± 12.44 ^a^	54.90 ± 8.10 ^a^	45.55 ± 4.10 ^b^	<0.001
Diagonal	66.46 ± 15.52 ^a^	66.57 ± 6.27 ^a^	57.69 ± 5.60 ^b^	<0.001
Heel–sole	72.86 ± 16.94 ^a^	74.89 ± 8.21 ^a^	65.88 ± 10.61 ^b^	<0.001
Dew claw	39.62 ± 12.04 ^a,b^	41.83 ± 6.23 ^a^	34.95 ± 5.21 ^b^	0.001
Anisodactylia Front Right (mm)	3.31 ± 3.17	5.10 ± 3.48	3.91 ± 2.95	0.143
Front left foot				
Medial claw lengths (mm)				
Dorsal	51.55 ± 12.18 ^a^	51.88 ± 7.05 ^a^	44.08 ± 4.78 ^b^	<0.001
Diagonal	65.60 ± 15.06 ^a^	63.04 ± 6.89 ^a^	56.71 ± 6.25 ^b^	<0.001
Heel–sole	67.17 ± 14.97 ^a,b^	70.67 ± 8.08 ^a^	65.51 ± 5.99 ^b^	0.043
Dew claw	37.72 ± 13.15 ^a,b^	39.62 ± 6.61 ^a^	33.76 ± 4.77 ^b^	0.005
Lateral claw lengths (mm)				
Dorsal	52.69 ± 11.50 ^a^	54.78 ± 6.66 ^a^	44.03 ± 3.52 ^b^	<0.001
Diagonal	65.51 ± 13.54 ^a^	65.22 ± 7.48 ^a^	57.44 ± 5.85 ^b^	<0.001
Heel–sole	71.63 ± 15.45 ^a^	73.05 ± 8.63 ^a^	67.55 ± 6.29 ^b^	0.007
Dew claw	40.35 ± 12.37 ^a^	40.86 ± 6.48 ^a^	34.72 ± 5.70 ^b^	0.008
Anisodactylia Front Left (mm)	4.07 ± 4.85	5.16 ± 3.52	3.02 ± 2.44	0.078

Claw lengths: Dorsal, the distance from the dorsal skin–horn junction (periople) to the apex of the toe; Diagonal, the distance from the apex of the toe to the skin–horn junction at the heel; Heel–sole, the length of the abaxial wall (sole) and bulb (heel) that are in contact with the floor surface from the top of the toe to the caudal end of the heel; Dewclaw, the length along dorsal wall from just below the coronary band to the end of the wall. Anisodactylia, the difference between lateral and medial dorsal claw lengths. ^a,b^ Mean values within the same row with different superscript letter are significantly different (*p* < 0.05).

**Table 3:** Claw lengths (mean ± SD) of medial and lateral claw of each rear foot in purebred sows of all three herds.

	Herd			
	A	B	C	*p*-Value
	*n* = 22	*n* = 19	*n* = 45	
Rear right foot				
Medial claw lengths (mm)				
Dorsal	67.35 ± 18.96 ^a^	58.34 ± 9.57 ^a^	44.38 ± 6.32 ^b^	<0.001
Diagonal	77.88 ± 19.70 ^a^	65.70 ± 9.37 ^a^	54.19 ± 5.64 ^b^	<0.001
Heel–sole	80.79 ± 23.44 ^a^	71.21 ± 10.37 ^a,b^	65.68 ± 7.42 ^b^	<0.001
Dew claw	43.33 ± 12.66 ^a^	42.32 ± 10.19 ^a^	35.67 ± 6.81 ^b^	0.002
Lateral claw lengths (mm)				
Dorsal	60.02 ± 17.60 ^a^	61.41 ± 11.30 ^a^	47.13 ± 8.22 ^b^	<0.001
Diagonal	73.05 ± 17.35 ^a^	72.79 ± 11.50 ^a^	56.96 ± 9.21 ^b^	<0.001
Heel–sole	81.29 ± 22.88 ^a^	80.73 ± 12.44 ^a^	65.26 ± 9.05 ^b^	<0.001
Dew claw	47.44 ± 16.00 ^a^	43.80 ± 11.30 ^a^	35.64 ± 6.99 ^b^	<0.001
Anisodactylia rear right (mm)	11.96 ± 11.86 ^a^	6.83 ± 4.24 ^a,b^	5.57 ± 5.06 ^b^	0.016
Rear left foot				
Medial claw lengths (mm)				
Dorsal	66.14 ± 14.85 ^a^	58.17 ± 11.23 ^a^	45.45 ± 5.73 ^b^	<0.001
Diagonal	77.96 ± 18.15 ^a^	65.60 ± 11.47 ^a^	53.70 ± 7.31 ^b^	<0.001
Heel–sole	79.43 ± 20.25 ^a^	72.36 ± 12.18 ^a^	64.38 ± 8.09 ^b^	<0.001
Dew claw	47.23 ± 17.66 ^a^	41.22 ± 8.90 ^a,b^	36.10 ± 7.03 ^b^	0.006
Lateral claw lengths (mm)				
Dorsal	60.83 ± 15.05 ^a^	62.62 ± 15.05 ^a^	47.31 ± 6.74 ^b^	<0.001
Diagonal	73.30 ± 16.49 ^a^	71.81 ± 16.37 ^a^	57.00 ± 8.62 ^b^	<0.001
Heel–sole	78.13 ± 20.70 ^a^	81.08 ± 15.55 ^a^	67.51 ± 9.32 ^b^	<0.001
Dew claw	45.63 ± 15.94 ^a^	42.01 ± 7.95 ^a^	35.40 ± 7.00 ^b^	<0.001
Anisodactylia rear left (mm)	8.69 ± 9.10 ^a^	5.91 ± 4.98 ^a,b^	4.07 ± 3.55 ^b^	0.041

Claw lengths: Dorsal, the distance from the dorsal skin–horn junction (periople) to the apex of the toe; Diagonal, the distance from the apex of the toe to the skin–horn junction at the heel; Heel–sole, the length of the abaxial wall (sole) and bulb (heel) that are in contact with the floor surface from the top of the toe to the caudal end of the heel; Dewclaw, the length along dorsal wall from just below the coronary band to the end of the wall. Anisodactylia, the difference between lateral and medial dorsal claw lengths. ^a,b^ Mean values within the same row with different superscript letter are significantly different (*p* < 0.05).

**Table 4:** Coefficients of correlation between the maximum temperature of each foot at four anatomical regions and the lengths of respective medial and lateral claws of purebred sows of all three herds (*n* = 86).

	Medial Claw Lengths			Lateral Claw Lengths			
	Dorsal	Diagonal	Heel–Sole	Dorsal	Diagonal	Heel–Sole	Anisodactylia
Max IRT1							
Front right foot	0.322 **	0.364 **	0.326 **	0.320 **	0.389 **	0.321 **	0.041
Front left foot	0.293 **	0.380 **	0.326 **	0.307 **	0.426 **	0.324 **	0.112
Rear right foot	0.290 **	0.288 **	0.206	0.287 **	0.322 **	0.267 *	0.228 *
Rear left foot	0.282 **	0.323 **	0.235 *	0.301 **	0.322 **	0.254 *	0.270 *
Max IRT2							
Front right foot	0.328 **	0.370 **	0.348 **	0.348 **	0.399 **	0.360 **	0.075
Front left foot	0.278 **	0.366 **	0.305 **	0.292 **	0.429 **	0.333 **	0.065
Rear right foot	0.285 **	0.281 **	0.225 *	0.267 *	0.311 **	0.270 *	0.247 *
Rear left foot	0.269 *	0.305 **	0.218 *	0.300 **	0.309 **	0.252 *	0.268 *
Max IRT3							
Front right foot	0.329 **	0.349 **	0.332 **	0.332 **	0.383 **	0.358 **	−0.010
Front left foot	0.292 **	0.407 **	0.340 **	0.299 **	0.450 **	0.358 **	0.077
Rear right foot	0.352 **	0.348 **	0.265 *	0.312 **	0.358 **	0.324 **	0.237 *
Rear left foot	0.224 *	0.274 **	0.170	0.300 **	0.306 **	0.245 *	0.176
Max IRT4							
Front right foot	0.300 **	0.327 **	0.308 **	0.332 **	0.381 **	0.303 **	0.05
Front left foot	0.322 **	0.431 **	0.336 **	0.317 **	0.435 **	0.375 **	0.112
Rear right foot	0.390 **	0.369 **	0.312 **	0.384 **	0.406 **	0.368 **	0.263 *
Rear left foot	0.360 **	0.395 **	0.339 **	0.343 **	0.354 **	0.342 **	0.260 *

Max IRT1: Maximum temperature at the joint of carpus in front legs or tarsus in rear legs; Max IRT2: Maximum temperature at the upper metacarpus or metatarsus area. Max IRT3: Maximum temperature at the lower metacarpus or metatarsus area; Max IRT4: Maximum temperature at the phalanges of each foot. Claw lengths: Dorsal, the distance from the dorsal skin–horn junction (periople) to the apex of the toe; Diagonal, the distance from the apex of the toe to the skin–horn junction at the heel; Heel–sole, the length of the abaxial wall (sole) and bulb (heel) that are in contact with the floor surface from the top of the toe to the caudal end of the heel; Dewclaw, the length along dorsal wall from just below the coronary band to the end of the wall. Anisodactylia, the difference between lateral and medial dorsal claw lengths. ** Correlation is significant at the 0.01 level (2-tailed). * Correlation is significant at the 0.05 level (2-tailed).

## Data Availability

Data are available from corresponding author upon request.
